# N6‐methyladenosine‐mediated LINC01087 promotes lung adenocarcinoma progression by regulating miR‐514a‐3p to upregulate centrosome protein 55

**DOI:** 10.1002/kjm2.12879

**Published:** 2024-07-18

**Authors:** Xin Zhang, Dong‐Jie Wang, Li Jia, Wei Zhang

**Affiliations:** ^1^ Department of Respiratory The First Affiliated Hospital of Harbin Medical University Harbin China

**Keywords:** centrosome protein 55, LINC01087, lung adenocarcinoma, miR‐514a‐3p, RNA binding motif protein 15

## Abstract

Long noncoding RNAs are key players in the development of lung adenocarcinoma (LUAD). The present study elucidated the role of LINC01087 in LUAD development. Cell vitality and apoptosis were assessed by the CCK‐8 assay and flow cytometry, respectively. The transwell assay was adopted to evaluate cell migration and invasion. Levels of m^6^A modification of LINC01087 were determined using the methylated RNA binding protein immunoprecipitation assay. The interactions among LINC01087, miR‐514a‐3p, and centrosome protein 55 (CEP55) were evaluated using dual‐luciferase reporter, RNA immunoprecipitation, and RNA–RNA pull‐down assays. LINC01087 was highly expressed in LUAD, and its downregulation restrained cancer cell proliferation, migration, invasion, and epithelial–mesenchymal transition in vitro as well as tumor growth in a xenograft tumor model. Overexpression of miR‐514a‐3p inhibited malignant phenotypes in LUAD cells by inactivating RhoA/ROCK1 signaling via the suppression of CEP55 expression. Mechanistically, RBM15 increased the expression and mRNA stability of LINC01087 by mediating its m^6^A modification and LINC01087 induced CEP55 expression by sponging miR‐514a‐3p. RBM15‐induced LINC01087 upregulation accelerated LUAD progression by regulating the miR‐514a‐3p/CEP55/RhoA/ROCK1 axis, illustrating the potential of LINC01087 as a novel target for LUAD therapy.

**Abbreviations**
CCK‐8: Cell counting kit‐8
CEP55: Centrosome protein 55
IHC: Immunohistochemistry
LncRNAs: Long non‐coding RNAs
LUAD: Lung adenocarcinoma
m^6^A: N6‐methyladenosine
Me‐RIP: Methylated RNA binding protein immunoprecipitation
MiRNAs: MicroRNAs
Non‐coding RNA: Non‐coding RNAs
nts: Nucleotides
RBM15: RNA‐binding motif protein 15
RhoA: Ras homolog gene family, member A
ROCK1: Rho‐associated protein kinase1
RT‐qPCR: Real‐time quantitative polymerase chain reaction

## 1 INTRODUCTION

Lung cancer, a malignant tumor with high morbidity and mortality,^1^ accounts for 40%–55% of all lung malignancies.^2^ Prognosis remains dismal in patients with lung adenocarcinoma (LUAD) despite recent breakthroughs in therapeutic options,^3^ as patients with early‐stage LUAD are usually asymptomatic and cancer is diagnosed in advanced stage in most patients, who cannot be treated with surgery.^4^ Therefore, investigating LUAD etiology is critical to identify relevant diagnostic and therapeutic biomarkers.

Long noncoding RNAs (lncRNAs), which are longer than 200 nucleotides,^5^ have a major role in cancer development. As widely described, LINC01087 aids in the growth of several cancer types. For example, one study reported increased LINC01087 expression in thyroid cancer and demonstrated that its silencing reduced the aggressiveness of cancer cells.^6^ In another study, LINC01087 was significantly increased in breast cancer and its downregulation inhibited the malignant tendency of breast cancer.^7^ Notably, a previous study found that LINC01087 was upregulated in lung squamous cell carcinoma.^8^ However, the mechanistic role of LINC01087 in LUAD remains unclear.

MicroRNAs (miRNAs), noncoding RNAs of approximately 22 nucleotides, are engaged in a variety of biological activities. Dysregulation of miRNAs is a well‐established contributor to carcinogenesis.^9^ For example, Luo et al. demonstrated that the overexpression of miRNA‐144‐5p restricted LUAD cell proliferation and promoted apoptosis.^10^ Additionally, miR‐514a‐3p, formerly known as miR‐514, is a member of the miRNA cluster on chrXq27.3 and serves as a tumor suppressor in multiple cancer types.^11^ As evidence, miR‐514a‐3p expression was significantly reduced in renal cell carcinoma, and its overexpression suppressed cancer cell malignant behaviors.^12^ Notably, miR‐514a‐3p was significantly downregulated in cisplatin‐resistant lung cancer cells whereas its overexpression facilitated cisplatin sensitivity.^13^ However, the role of miR‐514a‐3p in LUAD remains unclear.

Importantly, lncRNAs function as competing endogenous RNAs (ceRNAs) by serving as miRNA sponges and affecting the binding of miRNAs to downstream targets.^14^ For example, the lncRNA DGCR5 promoted LUAD cell apoptosis by sponging miR‐22‐3p.^15^ Bioinformatics analysis revealed that LINC01087 had potential binding sites to miR‐514a‐3p. However, the interaction between LINC01087 and miR‐514a‐3p in the regulation of LUAD progression remains unclear.

Centrosome protein 55 (CEP55), also known as c10orf3 and FLJ10540, is an important factor involved in the regulation of mitotic termination and cytoplasmic division.^16^ In clinical studies, CEP55 overexpression has been reported in multiple cancer types, including lung cancer.^17^ Notably, increased CEP55 expression in tumor samples was associated with LUAD prognosis.^18^ Rho‐associated protein kinase 1 (ROCK1) is a key serine/threonine kinase downstream of Ras homolog gene family, member A (RhoA), and the activation of RhoA/ROCK1 signaling can promote the invasion and migration in various cancer types, including lung cancer.^19^ Moreover, several reports have indicated that Polo‐like kinase 1 (Plk1) can regulate CEP55 and RhoA/ROCK1 signaling, and there is an interaction between them.^20^, ^21^ We previously predicted CEP55 as a target of miR‐514a‐3p through the analysis of multiple bioinformatics databases. Therefore, we speculated that miR‐514a‐3p might inhibit the progression of LUAD by regulating the CEP55/RhoA/ROCK1 axis.

N6‐methyladenosine (m^6^A) modification, as a prominent RNA modification, is a key player in LUAD progression.^22^ m^6^A modification is regulated by m^6^A methyltransferases (writers), m^6^A‐binding proteins (readers), and m^6^A demethylases (erasers).^23^ As a critical component of the m^6^A methyltransferase complex, RNA binding motif protein 15 (RBM15) regulates m^6^A modification by binding with targets and recruiting other methyltransferase.^24^ RBM15 is a risk factor for cancer progression.^25^ Notably, one study reported that RBM15 overexpression was associated with poor survival in patients with LUAD.^26^ Numerous recent studies have reported that m^6^A modification alteration mediates changes in lncRNA expression during carcinogenesis.^27^ In addition, we identified multiple m^6^A modification sites in LINC01087 mRNA using bioinformatic prediction, raising the possibility that RBM15 may maintain the stability of LINC01087 in an m^6^A‐dependent manner to regulate LUAD development.

Based on multiple lines of evidence, we hypothesized that RBM15‐mediated induction of LINC01087 stability would accelerate LUAD development by sponging miR‐514a‐3p to upregulate CEP55 and then to activate RhoA/ROCK1 signaling pathway. The present study identifies key molecules such as LINC01087 and miR‐514a‐3p that play important roles in the development of LUAD. Our study is expected to provide a theoretical basis for expanding the therapeutic framework for LUAD.

## 2 MATERIALS AND METHODS

### 2.1 Cell culture

The human normal bronchial epithelial cell line BEAS‐2B and the LUAD cell lines (H1975, A549, Calu‐3, and H358) were purchased from the American Type Culture Collection (VA, USA). The LUAD cell line PC‐9 was purchased from iCell (Shanghai, China). All cells were cultured in Dulbecco's modified Eagle's medium (DMEM; Gibco, MD, USA) containing 10% fetal bovine serum (FBS; Gibco) with 5% CO_2_ at 37°C.

### 2.2 Transfection

The full‐length CEP55 sequence was cloned into the pcDNA3.1 vector (GenePharma, Shanghai, China) to generate the CEP55 overexpression vector (oe‐CEP55), and pcDNA3.1 served as negative control. In brief, 1 μg of total RNA was used to synthesize full‐length CEP55 CDS, and the sequences were verified by DNA Sanger sequencing. A pair of specific primers, containing EcoR I和BamH I restriction enzyme cutting sites, were designed: forward 5′‐AAGCTTGGTACCGAGCTCGGATCCGCCACCATGTCTTCCAGAAGTACCAAAGATTTAAT‐3′ and reverse 5′‐CACTGTGCTGGATATCTGCAGAATTCCTACTTTGAACAGTATTCCACATGGACAAGC‐3′. Then, the target gene was then amplified. The amplification conditions were as follows: an initial denaturation at 95°C for 5 min; followed by 35 cycles of denaturation at 94°C for 20 s, annealing at 60°C for 30 s, and extension at 72°C for 2 min; lastly, extension at 72°C for 10 min. The PCR products were separated with 1.5% agarose gel electrophoresis, and the target fragments were retrieved and purified. The pcDNA3.1 vectors were cleaved by EcoR I and BamH I enzymes (Sangon, Shanghai, China). Then, the amplified fragment was linked to the linearized vector to complete the linking process between the target gene CEP55 and the pcDNA3.1 vector. The recombinant plasmids were transformed into *E. coli* DH5α competent cells for amplification. The recombinant plasmids were isolated sequenced by an ABI 377 DNA sequenator.

Short hairpin RNAs targeting LINC01087 (sh‐LINC01087: 5′‐GCAAGAATGTGGATTTATTTC‐3′) and RBM15 (sh‐RBM15: 5′‐AGGTGATAGTTGGGCATATAT‐3′), miR‐514a‐3p mimics (sense: 5′‐AUUGACACUUCUGUGAGUAGA‐3′, antisense: 5′‐UCUACUCACAGAAGUGUCAAU‐3′), miR‐514a‐3p inhibitor (5′‐UCUACUCACAGAAGUGUCAAU‐3′), and their negative controls (sh‐NC: 5′‐UUCUCCGAACGUGUCACGUTT‐3′; mimics NC: sense: 5′‐UCACAACCUCCUAGAAAGAGUAGA‐3′, antisense: 5′‐UCUACUCUUUCUAGGAGGUUGUGA‐3′; inhibitor NC: 5′‐UCUACUCUUUCUAGGAGGUUGUGA‐3′) were provided by GenePharma. Cells were plated 24 h before transfection in 24‐well culture plates at a density of 3 × 10^4^ cells per/well. Cells were switched to growth medium with 10% FBS without antibiotics before transfection. Then, 0.25 μg of overexpression plasmids, 2.5 pmol of shRNAs, or 25 nM miRNA mimics/inhibitor was first mixed with the reagent P3000 in Opti‐MEM™ Reduced Serum Medium and then added to Opti‐MEM™ Reduced Serum Medium containing Lipofectamine 3000 (Invitrogen, CA, USA). After 48 h, the transfected cells were harvested for RNA and protein isolation to determine the transfection efficiency. To stably knock down LINC01087 in LUAD cells, the lentivirus of sh‐LINC01087 and its negative control were constructed by Riobio (Guangdong, China). Cells (2 × 10^4^/well) were infected with the lentivirus combined with polybrene (5 μg/mL) for 24 h. Infected cells were collected for further investigation.

### 2.3 Real‐time quantitative polymerase chain reaction

For real‐time (RT) quantitative PCR (qPCR), total RNA was extracted using TRIzol (15596018CN; Thermo Fisher Scientific, MA, USA). The PrimeScript RT reagent kit (RR036A; Takara, Osaka, Japan) was used for first‐strand cDNA synthesis using 1 μg total RNA. RT‐qPCR was performed using the PowerUp SYBR Green master mix (A25743; Thermo Fisher Scientific) in an ABI7500 system. miRNAs were isolated using the miRNA isolation kit (9753Q; Takara) and quantified using the miRNA assay kit (638315; Takara). Glyceraldehyde 3‐phosphate dehydrogenase (GAPDH) and U6 were used as reference genes. Data were analyzed using the 2^−ΔΔCT^ method. RT‐qPCR was performed using triplicate and amplification conditions were as follows: an initial denaturation at 95°C for 5 min; followed by 35 cycles of denaturation at 94°C for 20 s, annealing at 59°C for 30 s, and extension at 72°C for 2 min; lastly, extension at 72°C for 10 min. The primer sequences are shown in Table 1.

**TABLE 1:** Primer information.

	Primer sequences (5′–3′)	Product length (bp)
LINC01087 (F)	ATGCTGTATTCTATGTCCTTTACCC	103
LINC01087 (R)	CTAATGCCACAAGCCTTTTCCT	
miR‐514a‐3p (F)	ATTGACACTTCTGTGAGTAGA	21
miR‐514a‐3p (R)	CAGTGCGTGTCTGGAGT	
CEP55 (F)	AGTAAGTGGGGATCGAAGCCT	168
CEP55 (R)	CTCAAGGACTCGAATTTTCTCCA	
GAPDH (F)	AGGTCGGTGTGAACGGATTTG	95
GAPDH (R)	GGGGTCGTTGATGGCAACA	
U6 (F)	CTCGCTTCGGCAGCACA	106
U6 (R)	AACGCTTCACGAATTTGCGT	

Abbreviations: CEP55, centrosome protein 55; GAPDH, Glyceraldehyde 3‐phosphate dehydrogenase.

### 2.4 Western blot analysis

After different treatments, cells were lysed using the RIPA buffer (P0013B; Beyotime), and protein concentrations were determined using a BCA kit (P0012; Beyotime). Next, 20 μg of protein samples were separated by sodium dodecyl sulfate–polyacrylamide gel electrophoresis using 10% gels and transferred to polyvinylidene fluoride membranes (Millipore, MA, USA). The membranes were blocked with 5% skim milk and incubated overnight with antibodies against E‐cadherin (1:1000; ab40772), vimentin (1:1000; ab92547), CEP55 (1:5000; ab170414), RhoA (1:5000; ab187027), ROCK1 (1:1000; ab134181), or GAPDH (1:2500; ab9485). The membranes were then incubated in the secondary antibody (1:6000; ab7090). All antibodies were purchased from Abcam (MA, USA). Protein bands were visualized using enhanced chemiluminescence (Beyotime), and band intensities using grayscale images were analyzed with ImageJ (The National Institutes of Health).

### 2.5 RhoA activity assay

Activity of the GTP‐bound RhoA was determined using the Rho activation assay kit (Cytoskeleton, CO, USA) according to the manufacturer's instructions. Briefly, cells were cultured in serum‐free medium containing ROCK1 and Rho GTPases inhibitors (Y‐27632 and Rhosin) for 24 h and lysed in the lysate buffer. Cell lysates were incubated overnight with the GST‐rhotekin‐RBD fusion protein. The levels of active GTP‐RhoA were determined with Western blot.

### 2.6 Cell counting kit‐8 assay

Cells were cultured at a density of 2 × 10^4^ cells/well in 24‐well plates for 24 h and incubated with the CCK‐8 solution (10 μL; E606335, Sangon, Shanghai, China) at 37°C for 3 h. Absorbance at 450 nm was determined using a microplate spectrophotometer (Thermo Fisher Scientific).

### 2.7 Apoptosis assay

Cells were grown at a density of 1 × 10^5^ cells/well in 6‐well plates for 12 h. Next, the cells were resuspended in Annexin‐binding buffer (500 μL; Beyotime) and stained for 10 min with Annexin V‐FITC and propidium iodide dye before analysis using flow cytometry (BD, NJ, USA).

### 2.8 Transwell migration/invasion assay

Cell migration and invasion assays were measured by trawnswell chamber. The upper chamber of the transwells (BD) was filled with 500 μL serum‐free DMEM containing 1 × 10^4^ cells, and the bottom chamber was filled with complete medium (1000 μL). After 12 h, the cells in the upper chamber were removed using cotton swabs. Next, the cells in the lower chamber were fixed with 4% paraformaldehyde and stained with 0.5% crystal violet. A light microscope (Thermo Fisher Scientific) was used to image cells. For cell invasion test, the upper chamber of the transwells was additionally precoated with Matrigel (Corning, NY, USA) at a ratio of 1:8.

### 2.9 Dual‐luciferase reporter assay

The Dual‐luciferase reporter assay was used to detect the binding relationship between LINC01087 /CEP55 and miR‐514a‐3p. In brief, 3′‐UTR fragments of LINC01087 (LINC01087‐WT: 5′‐ACAGGCAACAUGGAGUGUCAAU‐3′)/CEP55 (CEP55‐WT: 5′‐ACUACUAACAUUUUGCACUGUCAAA‐3′) containing miR‐514a‐3p binding sites or their corresponding mutated sequences (LINC01087‐MUT: 5′‐ACAGGCATGTUCGTCACAGTTU‐3′; CEP55‐MUT: 5′‐AGATGAGTGTUUUUCCTCACAGTTA‐3′) were amplified by PCR and introduced into the pGL3 reporter plasmids (Promega, WI, USA). The PCR products were subcloned into the XbaI site downstream of the stop codon in the PGL3‐Control firefly luciferase reporter vector. Cells were plated in 24‐well culture plates at a density of 3 × 10^4^ cells per/well. Then, cells were co‐transfected with LINC01087‐WT/CEP55‐WT or LINC01087‐MUT/CEP55‐MUT plasmids and miR‐514a‐3p mimics/inhibitor or mimics/inhibitor NC. Specifically, 0.25 μg of plasmids or 25 nM miRNA mimics/inhibitor was first mixed with the reagent P3000 in Opti‐MEM™ Reduced Serum Medium and then added to Opti‐MEM™ Reduced Serum Medium containing Lipofectamine 3000. Cells were washed with PBS, and 500 μL transfection mixture was added to each well. After incubating for 24 h, the luciferase activity was measured with Dual‐Luciferase Reporter Assay System (Promega). The Renilla luciferase activity is used for internal normalization.

### 2.10 Methylated RNA binding protein immunoprecipitation (me‐RIP) assay

The commercial Magna MeRIP™ m^6^A Kit (Millipore) was used to perform Me‐RIP assays according to the manufacturer's protocol. The cells were treated with m^6^A antibody (Abcam, ab151230, 2 μg/mg) or IgG antibody (Abcam, ab109489, 4 μg/mg) for 1 h and incubated with protein A/G magnetic beads for 1 h. Me‐RIP reaction mixture containing fragmented RNA, RNase buffer, and IP buffer antibody was incubated with beads that captured the m^6^A antibody for 2 h at 4°C. The RNAs were obtained from beads using elution buffer at 4°C with continuous shaking for 1 h. RNAs were then extracted and purified. Immunoprecipitated RNAs were analyzed using RT‐qPCR. In brief, the PrimeScript RT Reagent Kit (Takara) was used for first‐strand cDNA synthesis using 1 μg RNA. RT‐qPCR was performed in an ABI7500 system using the SYBR (Thermo Fisher Scientific). GAPDH was used as the reference gene. RT‐qPCR was performed in triplicate with the following protocol: 95°C for 2 min; followed by 35 cycles of denaturation at 94°C for 30 s, annealing at 60°C for 30 s, and extension at 72°C for 2 min; lastly, extension at 72°C for 10 min. The data were analyzed using 2^−ΔΔCT^ method. The abundance of m6A‐modified RNA determined by RT‐qPCR was then normalized to the input.

### 2.11 RNA binding protein immunoprecipitation (RIP) assay

The MagnaRIP RNA binding immunoprecipitation kit (Millipore) was used to determine whether LINC01087 was bound to RBM15 or Ago2. A total of 1 × 10^7^ cells were harvested and lysed in RIP lysis buffer containing protease inhibitor cocktail (Thermo Fisher Scientific) and RNA inhibitor (Beyotime), the supernatants were collected after centrifugation for 10 min at 13000 rpm. The supernatants were then incubated with IgG antibody (2 μg/mg; ab172730, Abcam), RBM15 antibody (3 μg/mg; ab70549, Abcam), or Ago2 antibody (4 μg/mg; ab186733, Abcam), and then incubated with protein A/G magnetic beads for 1 h. RNA was purified from the mRNA‐bead‐antibody complex and subjected to RT‐qPCR analysis. 1 μg RNA was used for cDNA synthesis. RT‐qPCR was performed in triplicate with the following protocol: 95°C for 2 min; followed by 35 cycles of denaturation at 94°C for 30 s, annealing at 55°C for 45 s, and extension at 72°C for 2 min; lastly, extension at 72°C for 10 min. GAPDH was used as the reference gene. The data were analyzed using 2^−ΔΔCT^ method. The abundance of protein‐bound RNA determined by RT‐qPCR was then normalized to the input.

### 2.12 RNA–RNA pull‐down assay

H1975 and A549 cells were incubated with biotinylated miR‐514a‐3p (Bio‐miR‐514a‐3p) or negative control vectors (Bio‐NC) for 2 h at 4°C to form miRNA‐RNA complexes. The formed complexes were captured using the magnetic beads conjugated with streptavidin (Thermo Fisher Scientific). Next, the complexes were eluted, and CEP55 mRNA expression was analyzed by RT‐qPCR. In brief, the PrimeScript RT Reagent Kit (Takara) was used for first‐strand cDNA synthesis using 1 μg RNA. RT‐qPCR was performed in triplicate with the following protocol: 95°C for 2 min; followed by 35 cycles of denaturation at 94°C for 30 s, annealing at 55°C for 30 s, and extension at 72°C for 2 min; lastly, extension at 72°C for 10 min. GAPDH was used as the reference gene. The data were analyzed using 2^−ΔΔCT^ method. The abundance of RNA‐bound RNA determined by RT‐qPCR was then normalized to the input.

### 2.13 mRNA stability assay

8 × 10^4^ cells were incubated with actinomycin D (5 mg/mL; Sigma‐Aldrich, MO, USA) for 0, 3, 6, 9, or 12 h. The cellular RNA was isolated, reverse‐transcribed to cDNA, and analyzed by RT‐qPCR.

### 2.14 Animal experiments

Fifteen 8‐week‐old male BALB/c nude mice (Charles River, Beijing, China) were randomly assigned to the control, sh‐NC, and sh‐LINC01087 groups (*n* = 5/group). Cells with stable LINC01087‐knockdown and those infected with lentivirus of negative control were trypsinized, washed, and resuspended in PBS. Next, 2 × 10^4^ cells in 0.2 mL volume were injected into the axilla of the right forelimb of mice. The tumor volumes were calculated as follows: V = L × W^2^/2 (L, length; W, width). After 30 d, all mice were euthanized and tumor tissues were collected. The animal experiments were approved by The First Affiliated Hospital of Harbin Medical University.

### 2.15 Immunohistochemistry

Following deparaffinization and antigen retrieval (Dako, CA, USA), tumor tissue sections were blocked in 5% bovine serum albumin and incubated overnight with antibodies against Ki67 (1:200; ab15580; Abcam) and CEP55 (1:400; ab170414; Abcam). The sections were then incubated with the secondary antibody (1:500; ab150077; Abcam) for 1 h, followed by staining with diaminobenzidine, counterstaining, dehydration, and mounting. Images of the sections were captured using an Olympus microscope (Tokyo, Japan).

### 2.16 Bioinformatics analysis

Differential expression of LINC01087 in LUAD tissues and adjacent normal tissues was predicted using starBase database (http://starbase.sysu.edu.cn). In addition, starBase database was utilized to predict the binding relationship between LINC01087 and miR‐514a‐3p. Moreover, the common target genes binding to miR‐514a‐3p were predicted by starBase, TargetScan (http://www.targetscan.org/vert_72/) and miRTar (http://mirtarbase.mbc.nctu.edu.tw/) databases. The SRAMP (http://www.cuilab.cn/sramp/) database was used to predict the m6A modification site of LINC01087.

### 2.17 Statistical analysis

All data were generated from at least three independent trials and expressed as means ± standard deviation. To ascertain the differences between groups, Student's *t* test or one‐way analysis of variance with Tukey's multiple comparisons test was utilized. A *p* value of <0.05 was considered statistically significant.

## 3 RESULTS

### 3.1 LINC01087 knockdown suppressed the proliferation, migration, invasion, and epithelial–mesenchymal transition of LUAD cells

**FIGURE 1:**
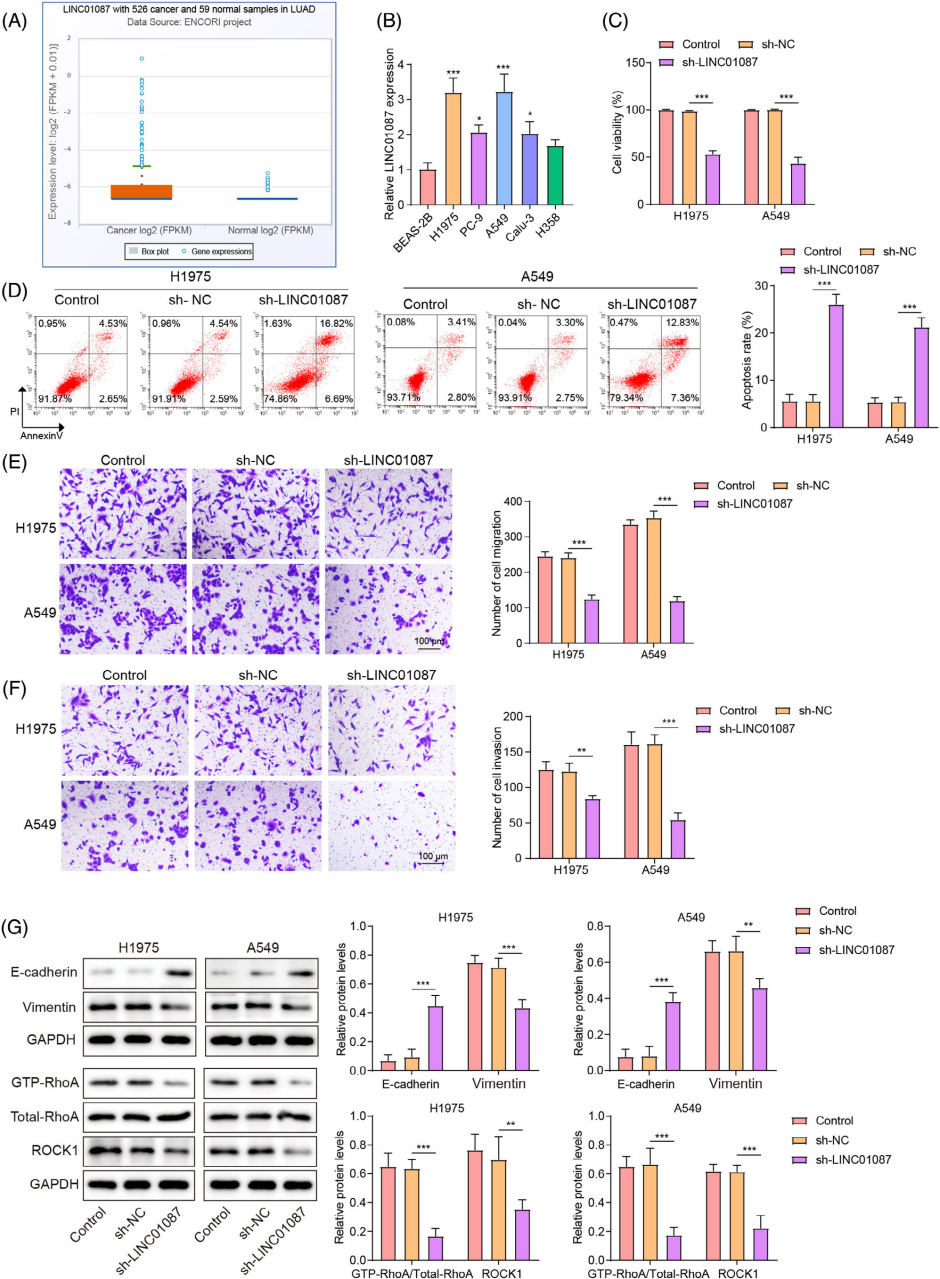
The effects of LINC01087 knockdown on lung adenocarcinoma (LUAD) cell proliferation, migration, invasion, and epithelial–mesenchymal transition (EMT). (A) LINC01087 expression in LUAD tissues and adjacent normal tissues was predicted using the starBase database. (B) Real‐time quantitative polymerase chain reaction was used to determine LINC01087 expression levels in LUAD cell lines (H1975, A549, PC‐9, Calu‐3, and H358 cells) and human normal bronchial epithelial cells (BEAS‐2B). H1975 and A549 cells were transfected with sh‐NC or sh‐LINC01087. (C) Cell viability was determined using the cell counting kit‐8 assay. (D) Apoptosis was determined by Annexin V‐PI staining via using flow cytometry. (E, F) Cell migration and invasion were assessed by the transwell assay. (G) Protein levels of EMT‐related proteins (E‐cadherin and vimentin) and Ras homolog gene family, member A (RhoA)/Rho‐associated protein kinase1 (ROCK1) signaling pathway markers (GTP‐RhoA, total‐RhoA, and ROCK1) were determined by Western blot. Data are presented as means ± standard deviation (SD). *N* = 3/group. All data were obtained from three independent replicates. GAPDH, Glyceraldehyde 3‐phosphate dehydrogenase. *p <0.05; **p <0.01; ***p <0.001.

The analysis using the starBase database predicted that LINC01087 expression was higher in LUAD tissues than in normal tissues (Figure 1A). Additionally, LINC01087 expression was significantly higher in five LUAD cell lines (H1975, A549, PC‐9, Calu‐3, and H358) than in BEAS‐2B cells (Figure 1B). LINC01087 expression was highest in H1975 and A549 cells, which were used for further evaluation. To investigate the role of LINC01087 in the regulation of LUAD progression, LINC01087 was knocked down in LUAD cells by transfecting sh‐LINC01087. As shown in Figure S1, sh‐LINC01087 transfection in LUAD cells significantly decreased LINC01087 expression. Other effects of LINC01087 silencing included observably reduced cell viability (Figure 1C), significant induction of LUAD cell apoptosis (Figure 1D), and decreased LUAD cell migration and invasion (Figure 1E,F). Furthermore, LINC01087 knockdown induced the levels of the epithelial cell marker E‐cadherin and decreased the levels of the mesenchymal cell marker vimentin (Figure 1G). Finally, LINC01087 knockdown led to reductions in GTP‐RhoA and ROCK1 levels in LUAD cells (Figure 1G). Collectively, these data indicated that LINC01087 facilitated malignant phenotypes and epithelial–mesenchymal transition (EMT) by activating the RhoA/ROCK1 signaling in LUAD cells.

### 3.2 RBM15 maintained the stability of LINC01087 in an m^6^A‐dependent manner

**FIGURE 2:**
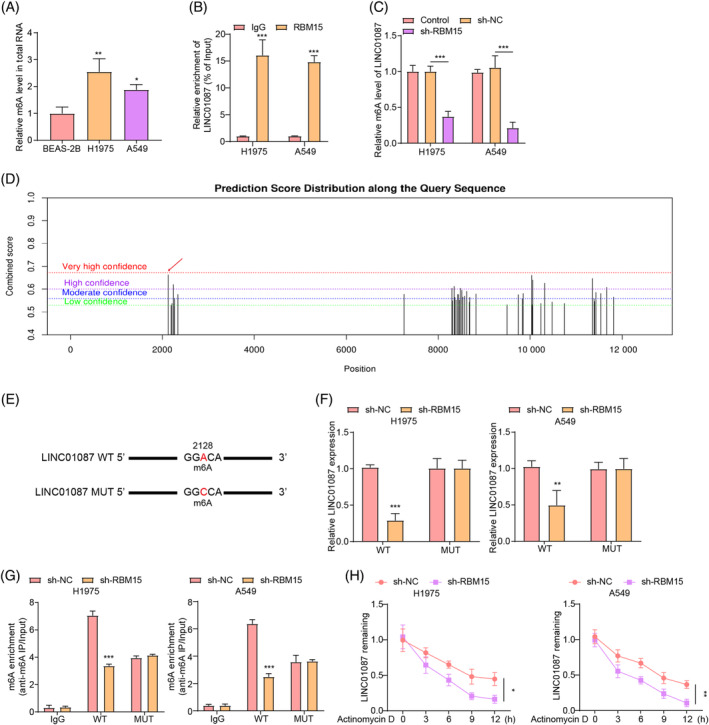
The effects of RNA‐binding motif protein 15 (RBM15) on the m^6^A modification level and stability of LINC01087. (A) Total m^6^A modification levels in lung adenocarcinoma (LUAD) cells and human bronchial epithelial cells were determined using the methylated RNA binding protein immunoprecipitation (Me‐RIP) assay. (B) The binding relationship between RBM15 and LINC01087 was determined using the RNA immunoprecipitation (RIP) assay. RBM15 antibody was used for RIP assay to isolate RBM15‐bonded RNAs, followed by real‐time quantitative polymerase chain reaction (RT‐qPCR) using LINC01087 specific primers. (C) Levels of m^6^A modification in LINC01087 after RBM15 knockdown in LUAD cells were determined with the MeRIP assay. (D) m^6^A modification sites in LINC01087 were predicted using the SRAMP database. (E) Schematic diagram showing m^6^A modification site and synonymous mutation in LINC01087. (F) The expression level of LINC01087 in LUAD cells cotransfected with sh‐RBM15 and LINC01087‐WT/LINC01087‐MUT was determined by RT‐qPCR. (A–C) Mut, adenine residues substituted by cytosine. (G) Levels of m^6^A modification in LINC01087 in LUAD cells cotransfected with sh‐RBM15 and LINC01087‐WT or LINC01087‐MUT were determined using the Me‐RIP assay. (H) LINC01087 mRNA stability in LUAD cells after RBM15 knockdown was determined with the RNA stability assay. Data are presented as means ± SD. *N* = 3/group. All data were obtained from three independent replicates. *p <0.05; **p <0.01; ***p <0.001.

In eukaryotic cells, m^6^A modification is a prominent RNA modification, which has been shown to regulate the expression of lncRNAs during carcinogenesis.^27^ Therefore, we determined whether LINC01087 was regulated by m^6^A modification in LUAD cells. As shown in Figure 2A, the total m^6^A modification level was significantly higher in LUAD cells than in BEAS‐2B cells, indicating that m^6^A modification might play an important role in LUAD progression. RBM15 is an important component of the methyltransferase complex involved in m^6^A modification. As shown in Figure S2, the levels of RBM15 were higher in LUAD cells than in BEAS‐2B cells. Notably, LINC01087 was highly abundant in the RNA complex precipitated by the RBM15‐specific antibody using the RIP assay, indicating that RBM15 was bound to LINC01087 (Figure 2B). Then, RBM15 knockdown was subsequently induced in LUAD cells, and sh‐RBM15 transfection markedly reduced RBM15 expression in LUAD cells (Figure S1). Notably, LINC01087 m^6^A level in LUAD cells was significantly reduced by RBM15 silencing (Figure 2C). Additionally, our analysis using the SRAMP database predicted multiple m^6^A modification sites in LINC01087 (Figure 2D). The schematic diagram of m^6^A modification at 2128 site in LINC01087 and synonymous mutation in LINC01087 was presented in Figure 2E. As shown in Figure 2F, RBM15 knockdown decreased LINC01087 levels in the LINC01087‐WT group but had no impact on LINC01087 levels in the LINC01087 m^6^A mutation group. Additionally, Me‐RIP assay found that RBM15 knockdown reduced the enrichment of m^6^A antibody in LINC01087‐WT group but had no effect on the m^6^A level of LINC01087 in the LINC01087‐MUT group (Figure 2G). Moreover, LINC01087 mRNA stability in LUAD cells was reduced by RBM15 knockdown (Figure 2H). Taken together, these results indicated that RBM15 induced LINC01087 expression and mRNA stability in LUAD cells in an m^6^A‐dependent manner.

### 3.3 LINC01087 decreased miR‐514a‐3p expression in LUAD cells by sponging miR‐514a‐3p

**FIGURE 3:**
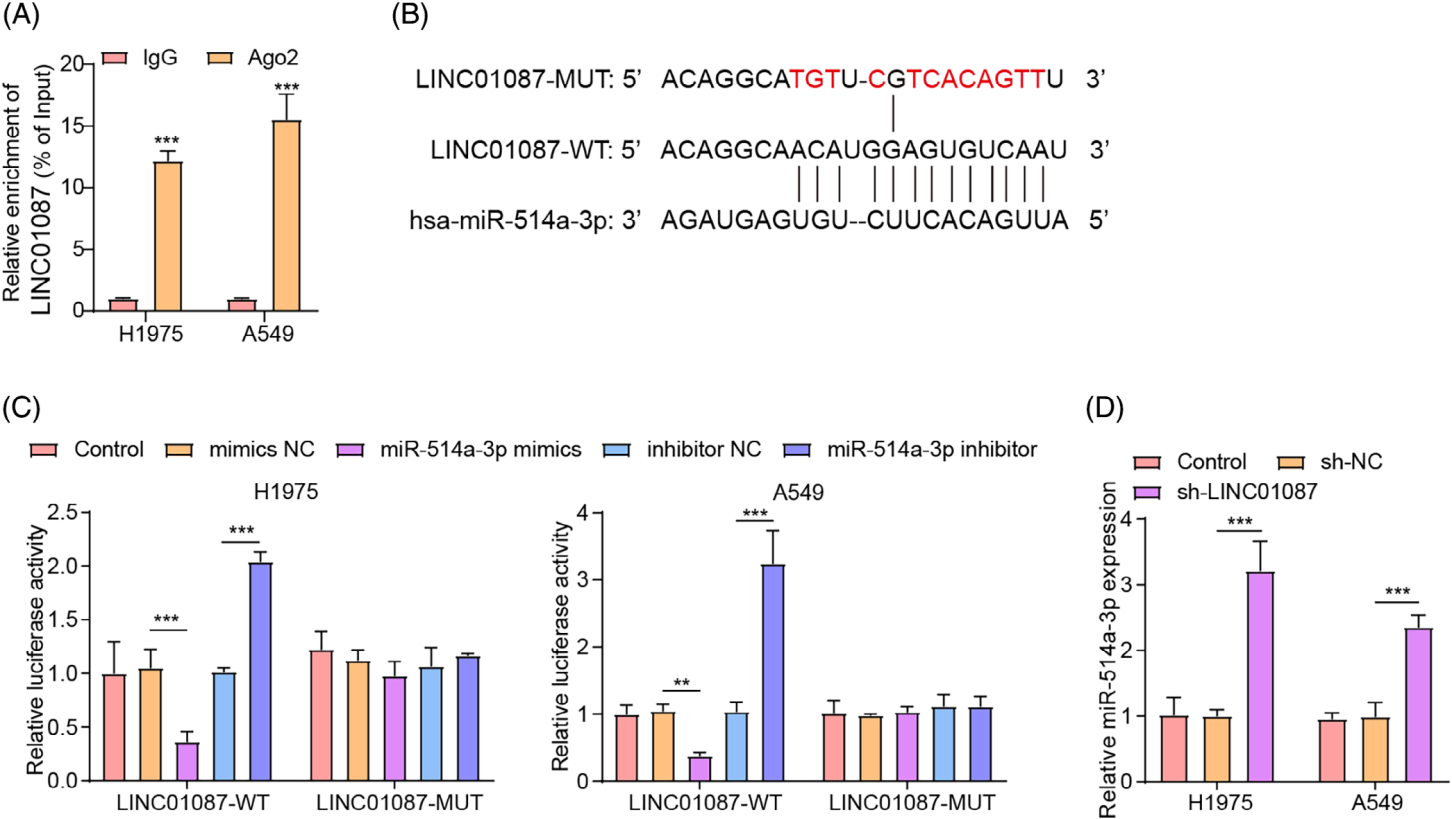
The target binding relationship between LINC01087 and miR‐514a‐3p in lung adenocarcinoma (LUAD) cells. (A) The binding relationship between LINC01087 and Ago2 was determined by RNA binding protein immunoprecipitation assay. (B) The potential binding site between LINC01087 and miR‐514a‐3p was predicted using the starBase database. (C) The interaction between LINC01087 and miR‐514a‐3p was determined using the dual‐luciferase reporter assay. (D) miR‐514a‐3p expression levels following sh‐NC or sh‐LINC01087 transfection in LUAD cells were determined using real‐time quantitative polymerase chain reaction. Data are presented as means ± SD. *N* = 3/group. All data were obtained from three independent replicates. **p <0.01; ***p <0.001.

As is well known, lncRNA can achieve its role in tumor biology by sponging miRNAs.^28^ By RIP assay, we found that LINC01087 was enriched in Ago2‐containing micronucleus proteins (Figure 3A), indicating that LINC01087 might function by sponging miRNAs. A ceRNA regulatory network for LINC01087 was constructed through the starBase, TargetScan, and miRTar databases, and it was discovered that miR‐514a‐3p was one of the important targets of LINC01087 (Figure S3). MiR‐514a‐3p is classical tumor suppressor.^29^ Notably, studies reported the reduced miR‐514a‐3p expression in cisplatin‐resistant non‐small cell lung cancer tissues and cells,^13^ indicating that miR‐514a‐3p might also play an antitumor role in LUAD. However, whether LINC01087 contributed to LUAD progression by regulating miR‐514a‐3p through its role as a ceRNA is unclear. To determine the role of miR‐514a‐3p in LUAD, we utilized miR‐514a‐3p knockdown and overexpression approaches by transfecting the miR‐514a‐3p inhibitor and mimics, respectively. As shown in Figure S1, miR‐514a‐3p expression levels in LUAD cells were dramatically reduced and increased following transfection with the miR‐514a‐3p inhibitor and the miR‐514a‐3p mimics, respectively. Using starBase database prediction, we found that LINC01087 harbored a potential binding site for miR‐514a‐3p (Figure 3B). As illustrated by the dual‐luciferase reporter assay, LINC01087 was directly bound to miR‐514‐3p (Figure 3C). Additionally, miR‐514‐3p expression levels in LUAD cells were markedly increased by LINC01087 knockdown (Figure 3D). Taken together, these results indicated that LINC01087 negatively regulated miR‐514a‐3p expression in LUAD cells by directly binding to miR‐514a‐3p.

### 3.4 Overexpression of miR‐514a‐3p inhibited the proliferation, migration, invasion, and EMT of LUAD cells

**FIGURE 4:**
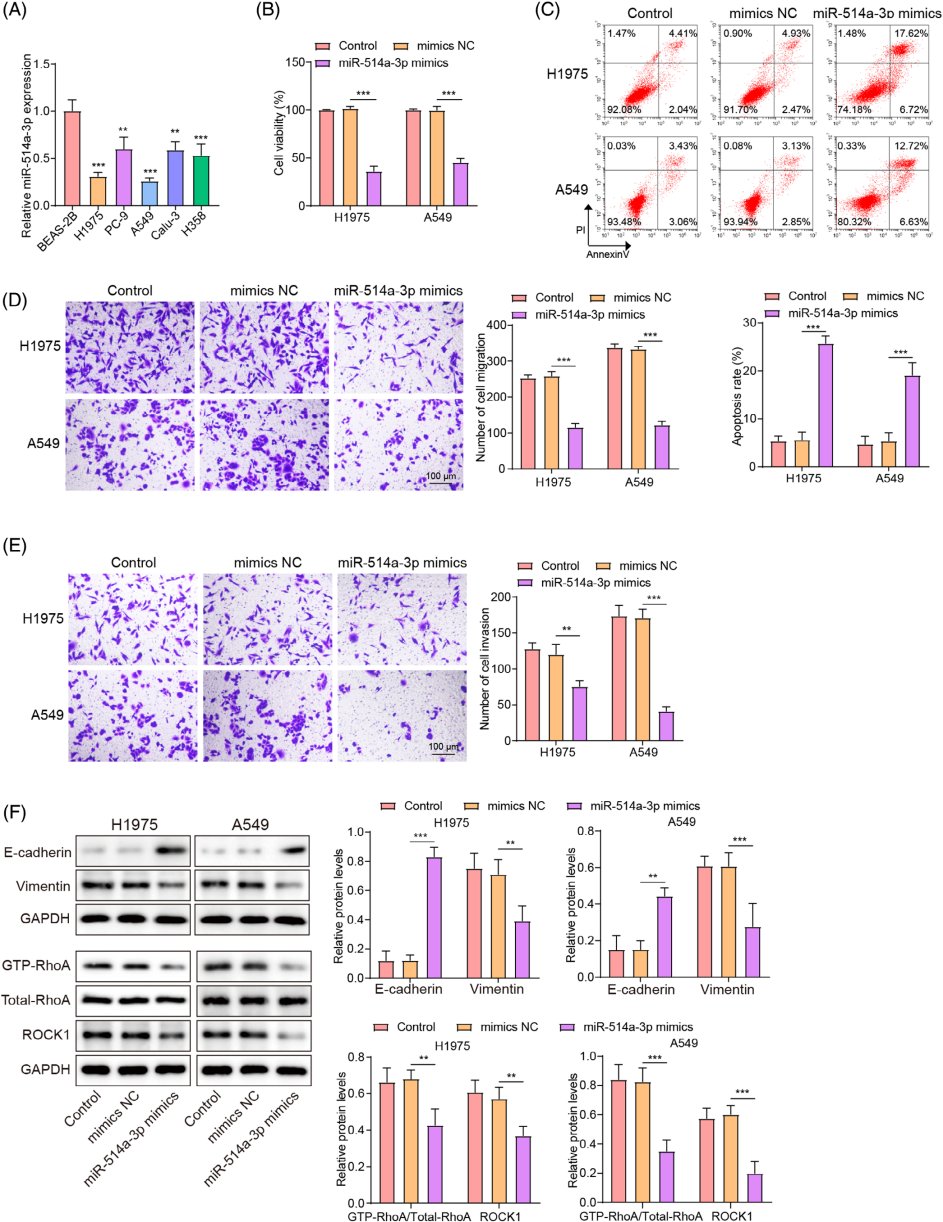
The effects of miR‐514a‐3p overexpression on lung adenocarcinoma (LUAD) cell proliferation, migration, invasion, and epithelial–mesenchymal transition. (A) Real‐time quantitative polymerase chain reaction was used to determine miR‐514a‐3p expression levels in LUAD cell lines (H1975, A549, PC‐9, Calu‐3, and H358) and human normal bronchial epithelial cells (BEAS‐2B). LUAD cells were transfected with miR‐514a‐3p mimics to overexpress miR‐514a‐3p. (B) LUAD cell viability was determined using the cell counting kit‐8 assay. (C) Apoptosis was determined by Annexin V‐PI staining with flow cytometer. (D, E) The transwell assay was used to analyze cell migration and invasion. (F) E‐cadherin, vimentin, GTP‐RhoA, total‐RhoA, and Rho‐associated protein kinase1 (ROCK1) levels in cells were determined using Western blot. Data are presented as means ± SD. *N* = 3/group. All data were obtained from three independent replicates. GAPDH, Glyceraldehyde 3‐phosphate dehydrogenase; RhoA, Ras homolog gene family, member A. **p <0.01; ***p <0.001.

As shown in Figure 4A, miR‐514a‐3p expression levels were lower in H1975, A549, PC‐9, Calu‐3, and H358 cells compared to BEAS‐2B cells, with the lowest levels detected in H1975 and A549 cells. Subsequent functional experiments revealed that miR‐514a‐3p overexpression significantly reduced LUAD cell viability (Figure 4B) and promoted apoptosis (Figure 4C). Additionally, miR‐514a‐3p overexpression significantly restrained LUAD cell migration and invasion (Figure 4D,E). Western blot revealed that miR‐514a‐3p overexpression induced E‐cadherin levels while reduced vimentin levels in LUAD cells (Figure 4F). Furthermore, miR‐514a‐3p upregulation dramatically reduced GTP‐RhoA and ROCK1 levels in LUAD cells (Figure 4F). Taken together, these results indicated that miR‐514a‐3p upregulation inhibited the malignant phenotypes in LUAD cells by inhibiting RhoA/ROCK1 signaling.

### 3.5 LINC01087 induced CEP55 expression by targeting miR‐514a‐3p in LUAD cells

**FIGURE 5:**
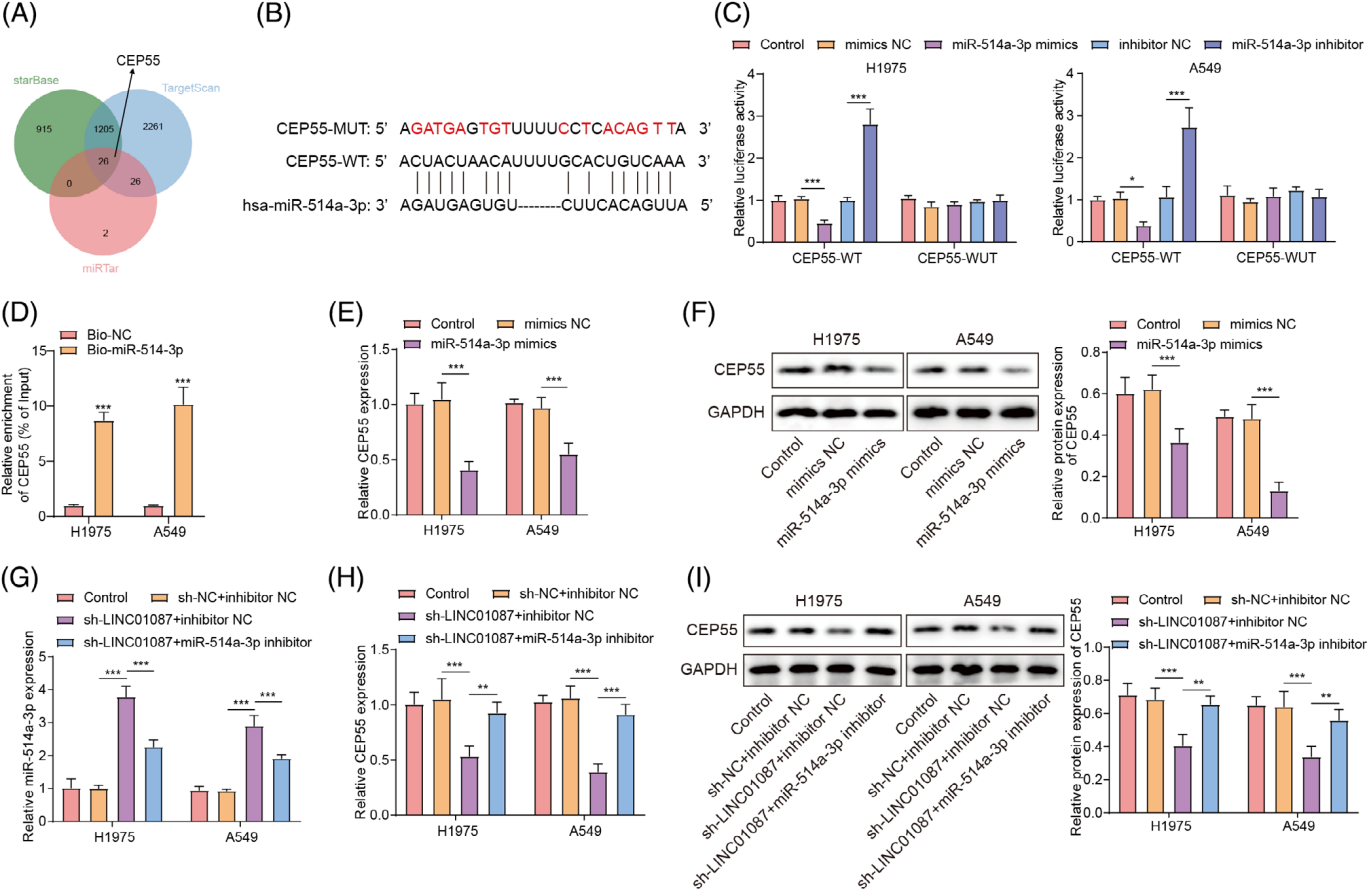
LINC01087 targeted miR‐514a‐3p to upregulate centrosome protein 55 (CEP55) expression in lung adenocarcinoma (LUAD) cells. (A) Common target genes binding to miR‐514a‐3p were predicted using the starBase, TargetScan, and miRTar databases. (B) Potential binding sites between miR‐514a‐3p and CEP55 were predicted using the starBase database. (C) The interaction between miR‐514a‐3p and CEP55 mRNA 3′UTR was determined using the dual‐luciferase reporter assay. (D) The interaction between miR‐514a‐3p and CEP55 mRNA 3′UTR was determined using RNA–RNA pull‐down assay with a biotinylated miR‐514a‐3p. RNA complexes bound to the miR‐514a‐3p were eluted, and the relative expression levels of CEP55 in the RNA complexes were determined using real‐time quantitative polymerase chain reaction (RT‐qPCR). (E, F) CEP55 expression levels in LUAD cells overexpressing miR‐514a‐3p were determined using RT‐qPCR and Western blot. (G) miR‐514a‐3p expression levels in LUAD cells following sh‐LINC01087 and miR‐514a‐3p inhibitor cotransfection were determined with RT‐qPCR. (H, I) CEP55 expression levels in LUAD cells after sh‐LINC01087 and miR‐514a‐3p inhibitor cotransfection were determined using RT‐qPCR and Western blot. Data are presented as means ± SD. *N* = 3/group. All data were obtained from three independent replicates. GAPDH, Glyceraldehyde 3‐phosphate dehydrogenase. *p <0.05; **p <0.01; ***p <0.001.

Using multiple bioinformatic predictions, we found that miR‐514a‐3p contained potential binding sites for CEP55 (Figure 5A,B). Dual‐luciferase reporter assay results demonstrated that miR‐514a‐3p overexpression significantly reduced while miR‐514a‐3p inhibition significantly increased the luciferase activity on CEP55‐WT group, and these changes disappeared when CEP55 was mutated, revealing that miR‐514a‐3p directly bound with CEP55 mRNA (Figure 5C). Additionally, the enrichment of CEP55 mRNA in biotinylated miR‐514a‐3p confirmed the interaction between CEP55 and miR‐514a‐3p (Figure 5D). Furthermore, CEP55 was significantly overexpressed in LUAD cells compared to BEAS‐2B cells (Figure S2), and CEP55 expression was significantly reduced by miR‐514a‐3p overexpression in LUAD cells (Figure 5E,F). In LUAD cells, LINC01087 knockdown induced miR‐514a‐3p expression, which was abrogated by miR‐514a‐3p inhibition (Figure 5G). LINC01087 knockdown also reduced the mRNA and protein levels of CEP55 in LUAD cells; these changes were reversed by miR‐514a‐3p knockdown (Figure 5H,I). These results suggested that LINC01087 induced CEP55 expression by targeting miR‐514a‐3p in LUAD cells.

### 3.6 miR‐514a‐3p overexpression inhibited the proliferation, migration, invasion, and EMT of LUAD cells by downregulating CEP55

**FIGURE 6:**
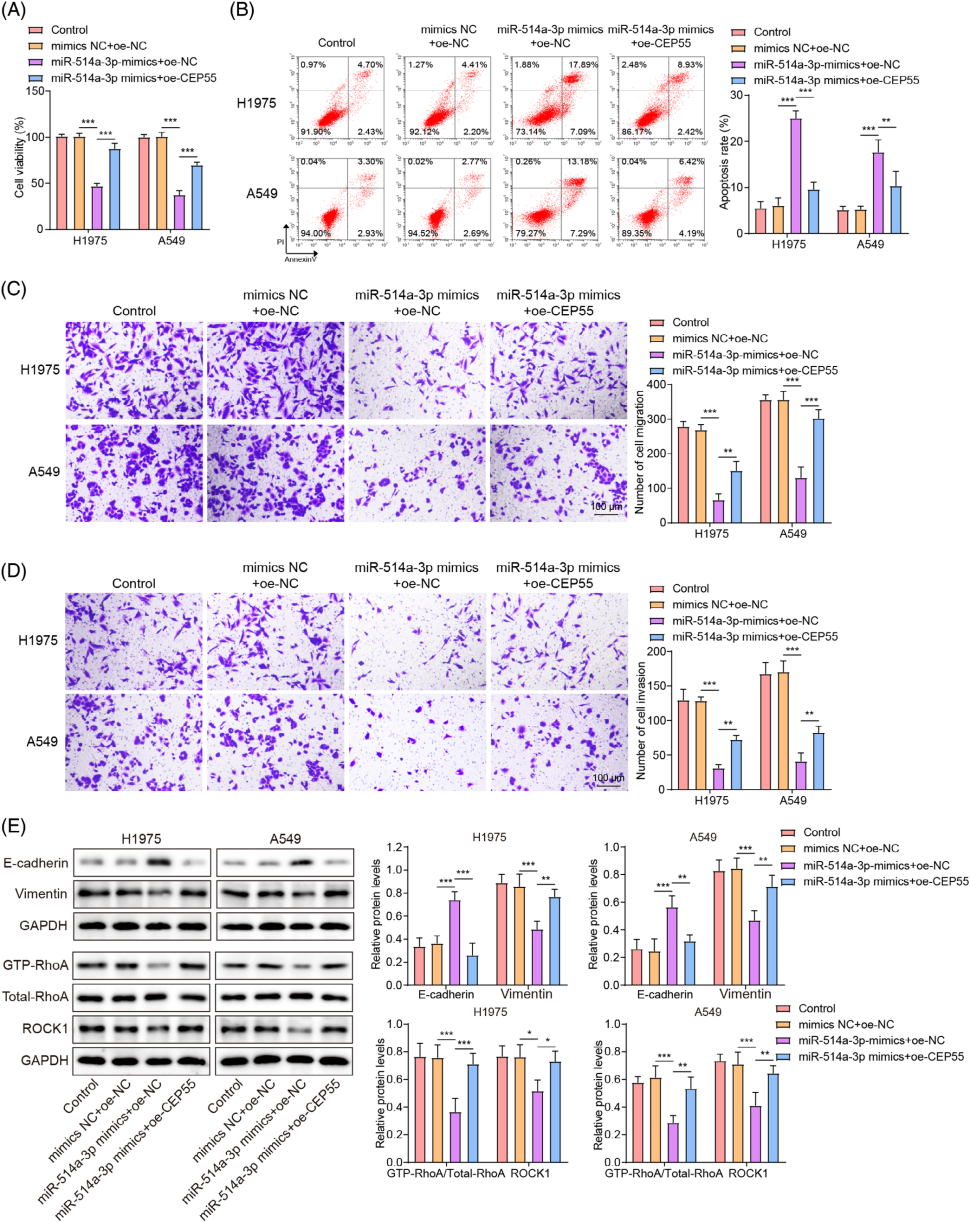
The inhibitory effects of miR‐514a‐3p overexpression on lung adenocarcinoma (LUAD) cell proliferation, migration, invasion, and epithelial–mesenchymal transition as well as Ras homolog gene family, member A (RhoA)/Rho‐associated protein kinase1 (ROCK1) signaling were abolished by centrosome protein 55 (CEP55) overexpression. LUAD cells were cotransfected with miR‐514a‐3p and CEP55 overexpression plasmids. (A) Cell viability was examined using cell counting kit‐8 assay. (B) Annexin V‐PI staining with flow cytometer was used to determine apoptosis. (C, D) Cell migration and invasion were determined using the transwell assay. (E) Western blot was used to determine E‐cadherin, vimentin, GTP‐RhoA, total‐RhoA, and ROCK1 levels in cells. Data are presented as means ± SD. *N* = 3/group. All data were obtained from three independent replicates. GAPDH, Glyceraldehyde 3‐phosphate dehydrogenase. *p <0.05; **p <0.01; ***p <0.001.

To elucidate the potential regulatory role of the miR‐514a‐3p/CEP55 axis in LUAD progression, miR‐514a‐3p and CEP55 overexpression approaches were used in LUAD cells. As illustrated in Figure S1, the inhibitory effect of miR‐514a‐3p overexpression on CEP55 expression levels in LUAD cells was reversed by CEP55 overexpression using oe‐CEP55. Additionally, CEP55 overexpression abrogated several effects mediated by miR‐514a‐3p overexpression in LUAD cells, including the inhibition of LUAD cell viability and promotion of apoptosis (Figure 6A,B) and the suppression of cell migration and invasion (Figure 6C,D). CEP55 overexpression also ameliorated the increases in E‐cadherin levels and the decreases in vimentin levels mediated by miR‐514a‐3p overexpression in LUAD cells (Figure 6E). CEP55 overexpression also abolished the reductions in GTP‐RhoA and ROCK1 levels in LUAD cells caused by miR‐514a‐3p overexpression (Figure 6E). In summary, these results indicated that miR‐514a‐3p overexpression suppressed the malignant phenotypes in LUAD cells by reducing CEP55 expression and consequently inactivating RhoA/ROCK1 signaling.

### 3.7 LINC01087 promoted the proliferation, migration, invasion, and EMT of LUAD cells by regulating the miR‐514a‐3p/CEP55 axis

**FIGURE 7:**
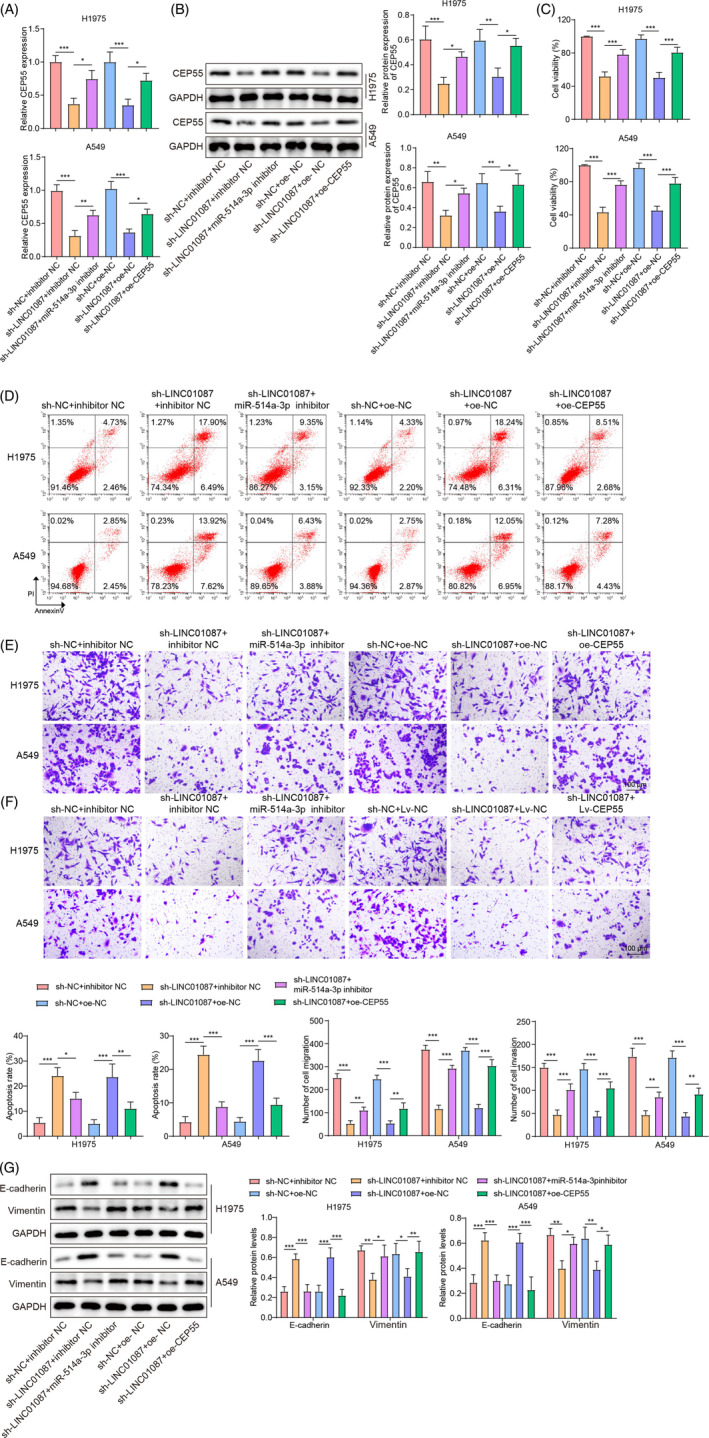
miR‐514a‐3p inhibition or centrosome protein 55 (CEP55) overexpression reversed the inhibitory effects of LINC01087 knockdown on lung adenocarcinoma (LUAD) cell proliferation, migration, invasion, and epithelial–mesenchymal transition. LUAD cells were cotransfected with miR‐514a‐3p knockdown/CEP55 overexpression and LINC01087 knockdown plasmids. (A, B) CEP55 expression levels in LUAD cells were determined with real‐time quantitative polymerase chain reaction and Western blot. (C) Cell viability was determined using cell counting kit‐8 assay. (D) Apoptosis was determined by flow cytometry. (E, F) The transwell assay was employed to determine cell migration and invasion. (G) E‐cadherin and vimentin levels in LUAD cells were determined using Western blot. Data are presented as means ± SD. *N* = 3/group. All data were obtained from three independent replicates. GAPDH, Glyceraldehyde 3‐phosphate dehydrogenase. *p <0.05; **p <0.01; ***p <0.001.

To investigate the role of the miR‐514a‐3p/CEP55 axis in LINC01087‐mediated promotion of malignant phenotypes in LUAD cells, miR‐514a‐3p and LINC01087 were knocked down whereas CEP55 was overexpressed. RT‐qPCR analysis confirmed significantly increased CEP55 expression levels in LUAD cells transfected with oe‐CEP55 (Figure S1). LINC01087 knockdown downregulated CEP55 expression in LUAD cells, whereas sh‐LINC01087‐mediated downregulation of CEP55 was abrogated by miR‐514a‐3p inhibition or CEP55 overexpression (Figure 7A,B). LINC01087 knockdown significantly inhibited LUAD cell viability and facilitated apoptosis, both of which were abrogated by miR‐514a‐3p inhibition or CEP55 overexpression (Figure 7C,D). Additionally, LUAD cell migration and invasion were significantly inhibited by LINC01087 knockdown; these effects were abolished by miR‐514a‐3p inhibition or CEP55 overexpression (Figure 7E,F). Consistently, LINC01087 knockdown led to increases in E‐cadherin levels and decreases in vimentin levels in LUAD cells; these changes were abrogated by miR‐514a‐3p inhibition or CEP55 overexpression (Figure 7G). These results indicated that LINC01087 facilitated malignant phenotypes by regulating the miR‐514a‐3p/CEP55 axis in LUAD cells.

### 3.8 LINC01087 knockdown inhibits LUAD tumor growth in vivo

**FIGURE 8:**
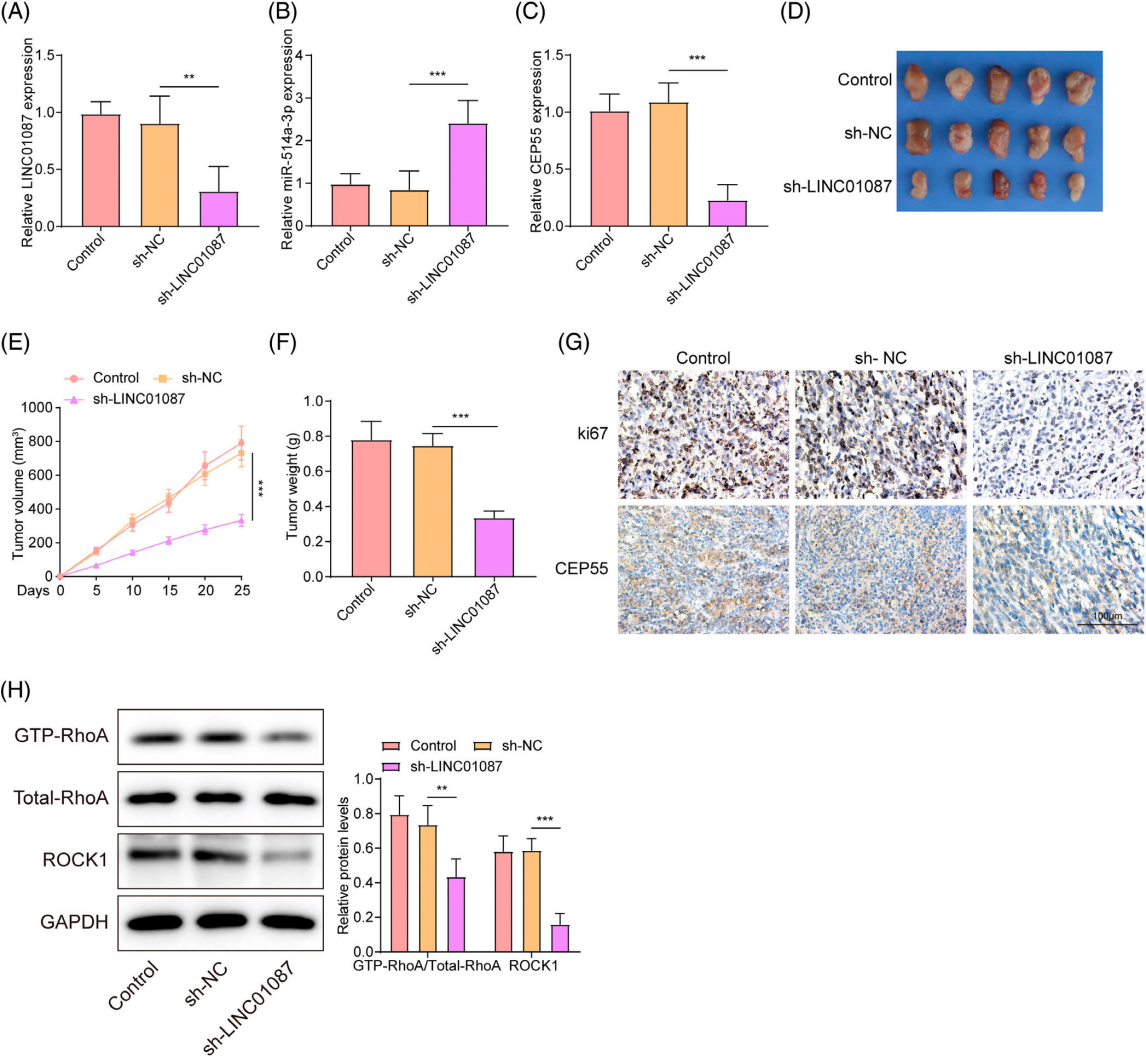
The effects of LINC01087 knockdown on lung adenocarcinoma (LUAD) tumor growth in vivo. Nude mice were injected with LUAD cells with stable LINC01087 knockdown. (A–C) LINC01087, miR‐514a‐3p, and centrosome protein 55 (CEP55) expression levels in tumor tissues were determined by real‐time quantitative polymerase chain reaction. (D–F) Tumor tissues were collected, and tumor volume and weight were measured. (G) Ki67 and CEP55 expression levels in tumor tissues were examined using immunohistochemistry. (H) Western blot was employed to determine GTP‐RhoA, total‐RhoA, and Rho‐associated protein kinase1 (ROCK1) levels in tumor tissues. Data are presented as means ± SD. GAPDH, Glyceraldehyde 3‐phosphate dehydrogenase; RhoA, Ras homolog gene family, member A. *N* = 5/group. **p <0.01; ***p <0.001.

Nude mice were injected with LUAD cells with stable LINC01087 knockdown to further examine the function of LINC01087 on LUAD tumor formation in vivo. As shown in Figure 8A–C, LINC01087 knockdown reduced LINC01087 and CEP55 expressions and increased miR‐514a‐3p expression in tumor tissues. Tumor growth was slower in mice injected with LUAD cells with stable LINC01087 knockdown than in those injected with LUAD cells without stable LINC01087 knockdown (Figure 8D–F). Additionally, the levels of the proliferation marker Ki67 and CEP55 in tumor tissues were markedly decreased by LINC01087 knockdown (Figure 8G). Furthermore, LINC01087 knockdown reduced GTP‐RhoA and ROCK1 levels in tumor tissues (Figure 8H). The above indicated that LINC01087 knockdown inhibited LUAD tumor growth in vivo by inactivating the RhoA/ROCK1 signaling.

## 4 DISCUSSION

LUAD is one of the most aggressive malignant tumors due to its rapid spread and poor prognosis.^30^ The majority of patients with LUAD are diagnosed after tumor metastasis and beyond the window of opportunity for surgical therapy, leading to a high mortality rate.^31^ Therefore, efforts should be focused on identifying effective biomarkers and understanding the pathogenesis of LUAD. In the present study, we demonstrated that RBM15‐induced LINC01087 upregulation promoted LUAD development by inhibiting miR‐514a‐3p expression, which in turn lead to the CEP55 upregulation and the subsequent activation of RhoA/ROCK1 signaling. Our findings provided a theoretical foundation for creating improved LUAD diagnosis and treatment options.

The critical function of lncRNAs in carcinogenesis has been widely described in recent decades. For example, the exogenous overexpression of lncRNA CASC2, whose expression was downregulated in LUAD, inhibited LUAD cell proliferation and facilitated apoptosis.^32^ Multiple studies have demonstrated that LINC01087 promotes carcinogenesis by regulating various cellular processes in a range of human cancers. For example, She et al.^7^ demonstrated that the expression of LINC01087 was elevated in breast cancer tissues and that its knockdown inhibited breast cancer cell proliferation, invasion, and migration via the miR‐335‐5p/ROCK1 axis. In addition, LINC01087 silencing suppressed thyroid cancer cell proliferation, invasion, and EMT via the miR‐135a‐5p/PPM1E axis.^6^ However, the function of LINC01087 in LUAD is unclear. Herein, our analyses revealed that LINC01087 was significantly upregulated in LUAD tissues and cells and that LINC01087 knockdown restrained proliferation, migration, invasion, and EMT and enhanced apoptosis in LUAD cells and reduced tumor development in vivo. Therefore, we provide the first evidence that LINC01087 exerts a carcinogenic role in LUAD.

The ceRNA regulation mechanism is a key mode of action for lncRNAs in cancer.^28^ We found that LINC01087 increased CEP55 expression by sponging miR‐514a‐3p in LUAD cells. In multiple cancer types, miR‐514a‐3p acts as a tumor suppressor.^12^ Notably, miR‐514a‐3p overexpression has previously been shown to reduce chemotherapy resistance in lung cancer^13^ and miR‐514a‐3p reversed the malignant phenotypes in LUAD cells promoted by TWIST1.^33^ Herein, the expression of miR‐514a‐3p, which was reduced in LUAD cells, was negatively regulated by LINC01087. Furthermore, miR‐514a‐3p overexpression inhibited malignant phenotypes in LUAD cells whereas miR‐514a‐3p downregulation abrogated the inhibitory effect of LINC01087 knockdown on phenotypes in LUAD cells. Collectively, these findings revealed that LINC01087 accelerated LUAD progression by sponging miR‐514a‐3p, which has not been reported to date. CEP55, abundantly expressed in proliferating cells, is located in the centrosome at the intermediate phase and forms homologous dimers to assist cell proliferation.^17^ CEP55 is widely expressed in cancers, notably lung cancer, and has been identified as a gene strongly linked to prognosis.^17^, ^34^ Notably, CEP55 is associated with poor prognosis in lung cancer.^17^ Our study revealed that CEP55 was upregulated in LUAD cells and LINC01087 upregulated CEP55 by sponging miR‐514a‐3p. We also demonstrated that CEP55 overexpression abrogated the inhibitory effects of LINC01087 knockdown or miR‐514a‐3p overexpression on the malignant phenotypes in LUAD cells, thereby verifying the ceRNA regulatory relationship involving LINC01087, miR‐514a‐3p, and CEP55 in LUAD. In addition, we also constructed the ceRNA regulatory network of LINC01087 (Figure S3), which has important significance for revealing new regulatory axes or therapeutic targets for LUAD treatment.

RhoA is a small GTP enzyme that interacts with the downstream effector ROCK1, a well‐known factor in cell motility.^35^ RhoA/ROCK1 signaling promotes cell migration, invasion, and metastasis in many cancer types, including lung cancer.^19^, ^36^ Notably, the relationship between CEP55 and RhoA/ROCK1 signaling has been discovered, with a focus on therapeutic consequences in cancer.^20^ In the present study, we demonstrate that RhoA/ROCK1 signaling acted as the downstream pathway of LINC01087/miR‐514a‐3p/CEP55 axis in LUAD cells. Collectively, LINC01087 increased CEP55 and activated RhoA/ROCK1 signaling by sponging miR‐514a‐3p to promote LUAD cell malignant behaviors. However, the exact mechanism by which CEP55 regulates RhoA/ROCK1 signaling remains unclear and warrants further investigation.

m^6^A modification is a highly abundant modification of mRNAs as well as noncoding RNAs.^37^ One study reported that m^6^A modifications were highly abundant in lncRNA TP53TG1 and that m^6^A modification‐induced overexpression of lncRNA TP53TG1 inhibited gastric cancer growth.^38^ Nevertheless, the effect of the m^6^A modification in regulating LINC01087 expression in LUAD remains unknown. Our study demonstrated that the overall m^6^A modification levels were substantially higher in LUAD cells than in normal bronchial epithelial cells. RBM15, as an m^6^A writer, adds m^6^A modifications to RNA molecules. The role of RMB15 in cancers has been widely studied. As proof, RBM15 knockdown suppressed colorectal cancer development by regulating the m^6^A levels in MYD88 mRNA.^39^ Herein, it turned out that RBM15 promoted LINC01087 mRNA stability and expression in LUAD cells, which has not been reported previously. What's more, m^6^A‐modified lncRNAs are regulated by m^6^A readers, which specific m^6^A recognition protein binds and stabilizes LINC01087 is worth further exploration.

Taken together, our analyses revealed that RBM15‐induced LINC01087 upregulation induced CEP55 expression and activated RhoA/ROCK1 signaling by sponging miR‐514a‐3p, thereby accelerating LUAD progression. The discovery of the role and regulatory mechanism of LINC01087 in LUAD has important guiding significance for the development of LUAD therapeutic drugs targeting LINC01087.

## CONFLICT OF INTEREST STATEMENT

All authors declare no conflict of interest.

## Supporting information


**Data S1.** Supporting information.

**Figure S1.** Detection of knockdown or overexpression effect of plasmids transfection. (A) Efficiency of LINC01087 knockdown was assessed by RT‐qPCR. (B, C) RBM15 expression levels in LUAD cells after sh‐NC or sh‐RBM15 transfection were tested by RT‐qPCR and Western blot. (D) miR‐514a‐3p expression in LUAD cells after miR‐514a‐3p inhibitor/mimics transfection was assessed using RT‐qPCR. (E, F) CEP55 expression levels in LUAD cells were determined using RT‐qPCR and Western blot. (G, H) CEP55 expression levels in LUAD cells after oe‐CEP55 or oe‐NC transfection were determined using RT‐qPCR and Western blot. The measurement data were presented as mean ± SD. *N* = 3 per group. All data were obtained from three replicate experiments. *p <0.05; **p <0.01; ***p <0.001.

**Figure S2.** Expression of RBM15 and CEP55 in different cell lines. (A, B) RBM15 expression levels in LUAD cells (H1975 and A549) and human bronchial epithelial cells were examined using RT‐qPCR and Western blot. (C) RT‐qPCR was adopted to detect CEP55 expression in LUAD cells (H1975, A549, PC‐9, Calu‐3, and H358) and human bronchial epithelial cells (BEAS‐2B). The measurement data were presented as mean ± SD. *N* = 3 per group. All data were obtained from three replicate experiments. *p <0.05; **p <0.01; ***p <0.001.

**Figure S3.** Construction of the ceRNA network based on LINC01087. LncRNA‐miRNA‐mRNA network was constructed according to prediction results from different databases. Starbase database was used to predict the miRNAs with targeted binding relationship to LINC01087, and the target genes binding to miR‐514a‐3p were predicted by Starbase, TargetScan, and miRTar databases.
