# Supplementary figures and images for: N6‐methyladenosine‐mediated LINC01087 promotes lung adenocarcinoma progression by regulating miR‐514a‐3p to upregulate centrosome protein 55
Source: Kaohsiung J Med Sci. 2024 Jul 18;40(9):801–18. doi: 10.1002/kjm2.12879 (PMC11895572; doi:10.1002/kjm2.12879)

**Sequencing result of CEP55(NM 001127182) -pcDNA3.1 (+) vector**


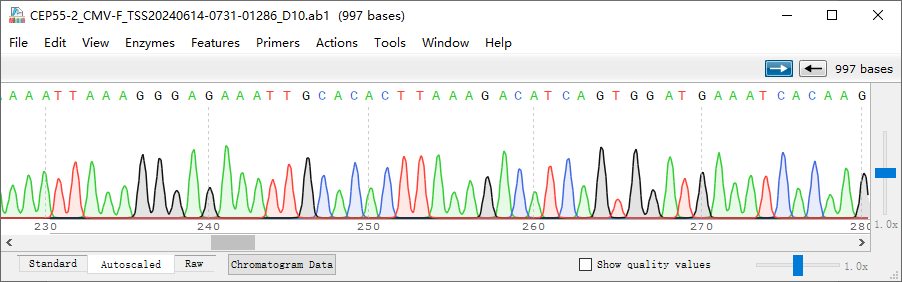

Supplement: Supplementary file 1 — Data S1. Supporting information. [file KJM2-40-801-s001.docx]

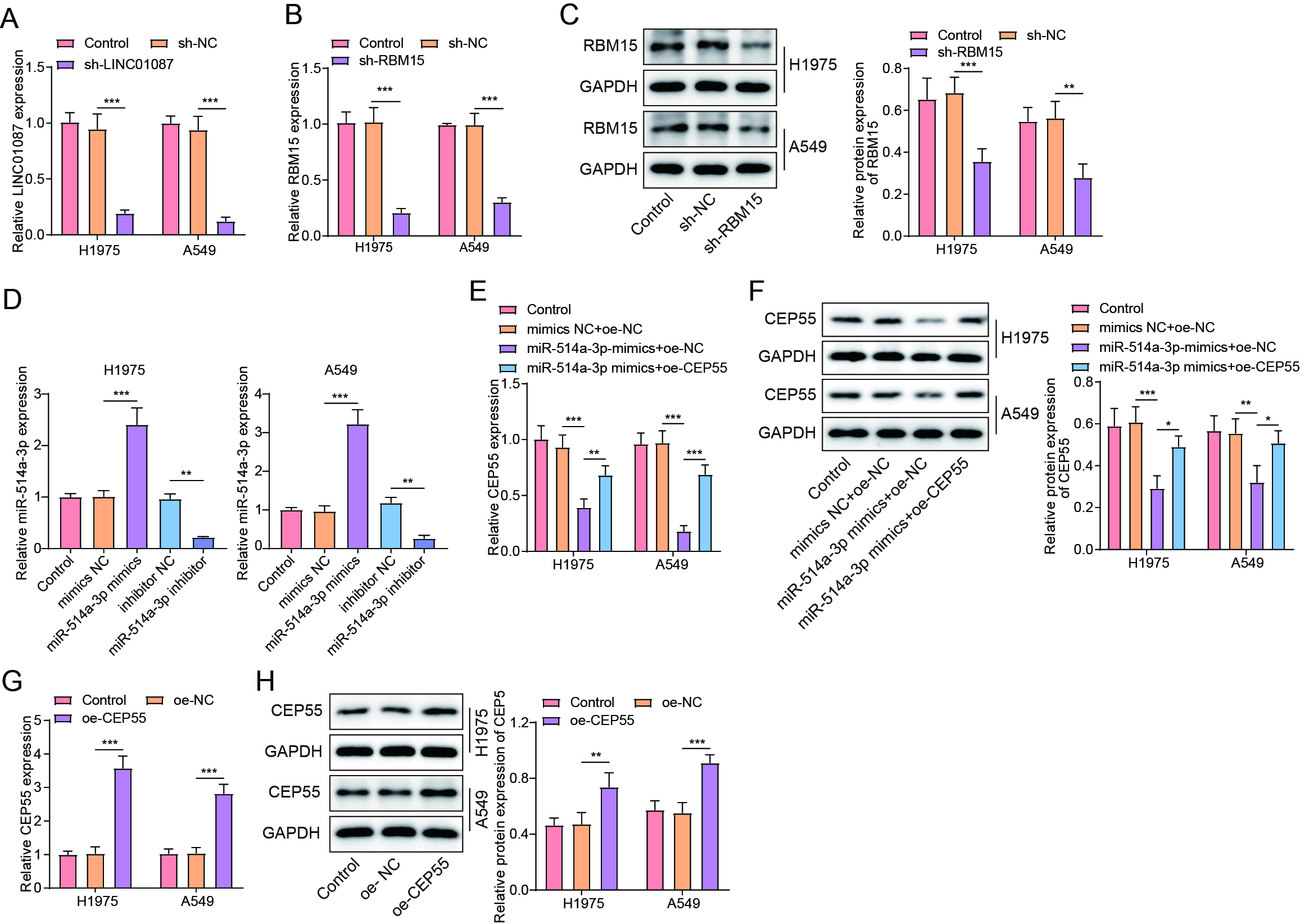

Supplement: Supplementary file 2 — Figure S1. Detection of knockdown or overexpression effect of plasmids transfection. (A) Efficiency of LINC01087 knockdown was assessed by RT‐qPCR. (B, C) RBM15 expression levels in LUAD cells after sh‐NC or sh‐RBM15 transfection were tested by RT‐qPCR and Western blot. (D) miR‐514a‐3p expression in LUAD cells after miR‐514a‐3p inhibitor/mimics transfection was assessed using RT‐qPCR. (E, F) CEP55 expression levels in LUAD cells were determined using RT‐qPCR and Western blot. (G, H) CEP55 expression levels in LUAD cells after oe‐CEP55 or oe‐NC transfection were determined using RT‐qPCR and Western blot. The measurement data were presented as mean ± SD. N = 3 per group. All data were obtained from three replicate experiments. *p <0.05; **p <0.01; ***p <0.001. [file KJM2-40-801-s004.tif]

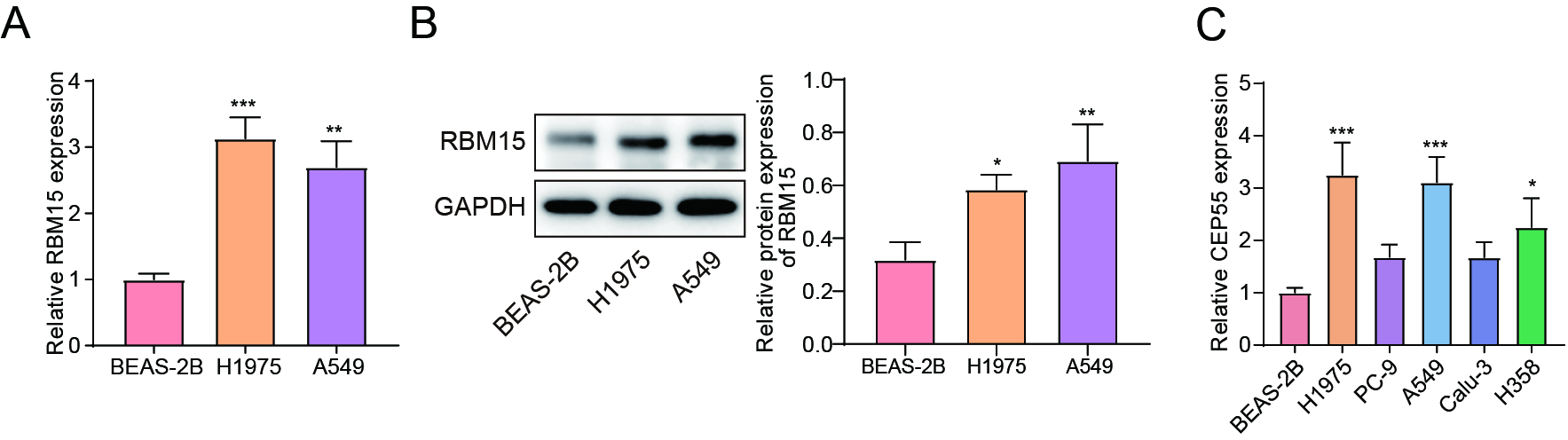

Supplement: Supplementary file 3 — Figure S2. Expression of RBM15 and CEP55 in different cell lines. (A, B) RBM15 expression levels in LUAD cells (H1975 and A549) and human bronchial epithelial cells were examined using RT‐qPCR and Western blot. (C) RT‐qPCR was adopted to detect CEP55 expression in LUAD cells (H1975, A549, PC‐9, Calu‐3, and H358) and human bronchial epithelial cells (BEAS‐2B). The measurement data were presented as mean ± SD. N = 3 per group. All data were obtained from three replicate experiments. *p <0.05; **p <0.01; ***p <0.001. [file KJM2-40-801-s002.tif]

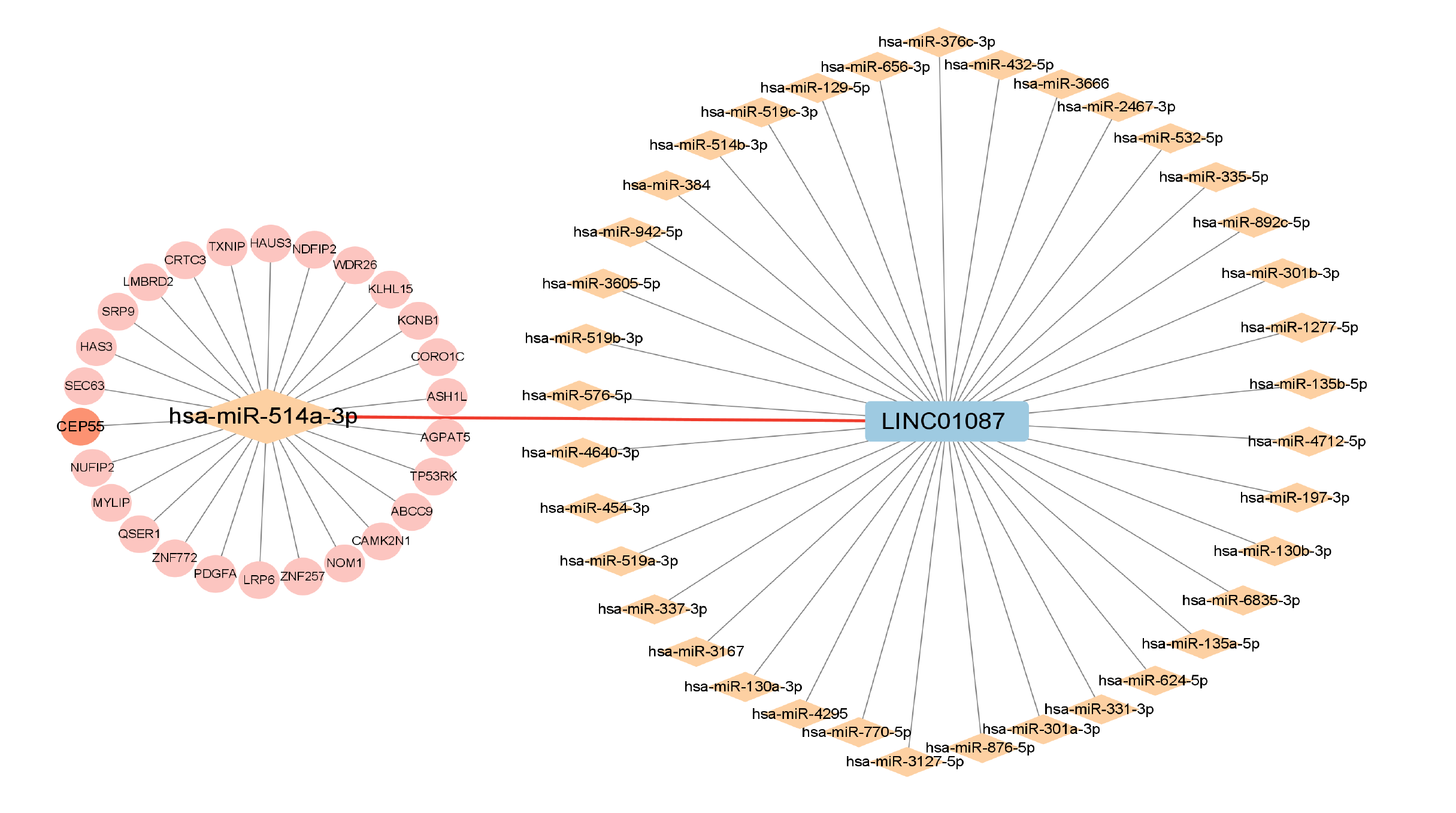

Supplement: Supplementary file 4 — Figure S3. Construction of the ceRNA network based on LINC01087. LncRNA‐miRNA‐mRNA network was constructed according to prediction results from different databases. Starbase database was used to predict the miRNAs with targeted binding relationship to LINC01087, and the target genes binding to miR‐514a‐3p were predicted by Starbase, TargetScan, and miRTar databases. [file KJM2-40-801-s003.tif]
